# The Caustive Factor in the Production of Otitis Media in Atlanta

**Published:** 1907-07

**Authors:** Claude A. Smith

**Affiliations:** President Fulton County Medical Association. Bacteriologist to City of Atlanta


### Journal-Record of Medicine

Successor to Atlanta Medical and Surgical Journal, Established 1855, and Southern Medical Record, Established 1870.

OWNED BY THE ATLANTA MEDICAL JOURNAL CO.

Published Monthly.

*Official Organ Fulton County Medical Society, State Examining Board, Presbyterian Hospital, Etc.*

VOL. IX. JULY, 1907. NO. 4

BERNARD WOLFF, M. D., EDITOR, 319-320 Prudential Bldg.

GEORGE BROWN, M. D., BUSINESS MANAGER, Business Office 1½-5½ S. Broad St.

E. G. BALLENGER, M. D., ASSOCIATE EDITOR AND ASSISTANT BUSINESS MANAGER.

### ORIGINAL COMMUNICATIONS.

### THE CAUSTIVE FACTOR IN THE PRODUCTION OF OTITIS MEDIA IN ATLANTA.

By Claude A. Smith, M.D., President Fulton County Medical Association. Bacteriologist to City of Atlanta.

About six years ago the writer began a series of examinations of cases of otitis media in Grady Hospital, and found in every instance that the condition was apparently due to a streptococcic infection. The first microscopical examinations indicated that the infective organism was an intera-cellular diplococcus. However, upon careful examination it was found that occasionally the organism would appear in chains of four or more cocci.

Growth upon culture media showed a typical streptococcus in each instance. The cases coming into the hospital were never seen before the drum membrane was ruptured, and it was therefore not possible to secure cuttures before this rupture had occurred and the pus had become contaminated with the other organisms present in the external auditory canal. Therefore, the first examination of the pus always showed quite a variety of bacteria.

At this time Dr. Roy was using 1-5000 bichloride of mercury solution for irrigation in these cases and it was found that after irrigating the ear every two or three hours for a day or so that all organisms would disappear from the pus with the exception of this streptococcus which was usually found inside the pus cells and in pairs with an occasional short chain formation. There were about eight cases of middle ear suppuration in the hospital at this time, and after irrigation with the bichloride this streptococcus was present in each case in pure culture.

These cases appeared in January and February, and in a few weeks all had cleared up. The following winter at about the same time cases of middle ear suppuration again appeared in the hospital and under the same procedure the same organism was isolated in each case. Since that time cases of middle ear suppuration have appeared in the hospital every winter, and in every case this organism has been isolated.

The infection is peculiar in that it almost invariably appears about the same time each year. Some years there have been not more than a half dozen cases in the hospital during the entire winter, and then again there have been as many as fifteen cases in the hospital at one time. Occasionally cases are seen at other seasons of the year, but this is the exception.

The disease appears to be mildly contagious. During one winter one of the nurses who had been nursing several of these cases developed a suppuration of the middle ear, and examination showed the same organism which was present in the cases which she had been nursing.

The attack is usually preceded by a cold or sore throat, but as the disease appears at the time of year when colds and sore throats are very common, this symptom is of uncertain significance. One of the first cases examined complained of slight tenderness about the joints, but this cleared up in a day or so, while the discharge from the ear continued for several days.

The amount of pain accompanying the condition is variable, some complain of excruciating ear-ache before the rupture of the drum, while others give a history of very little trouble.

When the drum first ruptures there is a profuse discharge, pale mucoid pus which gradually decreases in amount and ceases in about a week or ten days. One ear is usually involved, but occasionally both may be discharging at the same time.

The interference with the hearing is usually not very great, and entirely clears up in a few weeks or months.

As before stated, when first observed in the pus the organism appears to be in pairs, but further observation showed the organism growing in short chains of four or more cocci, and occasionally as many as twelve or more.

When grown on culture media, however, they produce chains of longer length. On solid media they produced small pearly pin-point colonies, and at 37 degrees centigrade reached their maximum growth in about 24 hours. In a few days the colonies appear ragged at the edges and lose their pearly appearance. It grows at the ordinary room temperature, and does not die out quickly.

The organism appears flattened on both sides, and has very little, if any, tendency to group itself in pairs in the chain formation. Occasionally one or two will appear with a greater diameter than the adjacent cocci. The organism decolorizes by Gram's method of staining, but stains readily by ordinary dyes. In bouillon there is no diffuse cloudiness, but flocculent masses collect in the bottom of the tube and occasionally on the sides. Microscopical examination shows these masses to consist of a net work of strepto cocci.

At first this organism was supposed to be the streptococcus pyogenes. However, its staining reaction did not appear to conform to this organism. Quite a variety of streptococci have been described from time to time by various investigators, and attempts have been made to classify these different streptococci. It is generally considered, however, that these streptococci found about the human body are types of this streptococcus pyogenes. In 1891 Von Lingelshein classified these streptococci as pathogenic and non-pathogenic. Under the pathogenic, he classified all pyogenic streptococci which grew into long chains and spoke of them as the streptococcus longus, and under this head he sub-classified the streptococcus murisepticus, and the streptococcus pyogenes and also the streptococcus erysipelatos. Under the head of non-pathogenic, he classified the streptoccoccus brevis. In classifying these, he speaks as follows:

"These cannot be distinguished one from the other in cultures in highly abluminous media (pus, blood-serum), but present constant differences when cultivated in bouillon. The decisive characterstics in this medium are: macroscopic, the cloudiness of the medium; microscopic, the length of the chains."

Since this classification some investigators describe streptococci which conform to Von Lingelshein's streptococcus brevis, but they call the organism streptococcus pyogenes.

Goadby describes the streptococcus brevis which he finds constantly in the mouths of people both in health and disease. While in the mouth this organism appears usually in diplococcic form, but grows in chains or culture medium. This streptococcus brevis resembles closely the organism which we have isolated from these cases of suppuration of the middle ear. With the exception of slight variations in growth on culture media it appears identical, except that the organism that we have isolated does not stain by Gram.

Von Lingelshein also states that the brevis uniformly clouds the bouillon; the organism we have isolated leaves the bouillon clear, forming a deposit in the bottom of the tube, thus conforming to his description of the growth of the streptococcus longus.

From time to time various investigators have isolated streptococci from cases of otitis media. (1.) Levy and Shrader found a staphylococcus albus in pure cultures in three cases out of ten which paracentesis was performed. Netter found streptococcus pyogenes thirteen times in eighteen cases. Scheibe obtained the streptococcus pyogenes, diplococcus pneumoniae, staphylococcus aurius, etc. Bordoni-Uffreduzzi and Gradenigro found the diplococcus pneumoniæ to be the most common organism in middle ear suppuration.

The fact that we have found only one organism in suppuration of the middle ear is probably a coincidence, but that we have found the same organism in so many cases indicates that it is the most common factor in the production of otitis media in our section of the country.

